# Digital twins for cardiac electrophysiology: state of the art and future challenges

**DOI:** 10.1007/s00399-024-01014-0

**Published:** 2024-04-12

**Authors:** Matthijs J. M. Cluitmans, Gernot Plank, Jordi Heijman

**Affiliations:** 1 Department of Cardiology, Cardiovascular Research Institute Maastricht, Faculty of Health, Medicine, and Life Sciences, Maastricht University, Maastricht, The Netherlands; 2 Gottfried Schatz Research Center, Division of Medical Physics & Biophysics, Medical University of Graz, Neue Stiftingtalstraße 6, 8010 Graz, Austria

**Keywords:** Arrhythmias, Computer model, Virtual arrhythmia risk prediction, Precision medicine, Simulation, Herzrhythmusstörungen, Computermodelle, Vorhersage des virtuellen Arrhythmierisikos, Präzisionsmedizin, Simulation

## Abstract

Cardiac arrhythmias remain a major cause of death and disability. Current antiarrhythmic therapies are effective to only a limited extent, likely in large part due to their mechanism-independent approach. Precision cardiology aims to deliver targeted therapy for an individual patient to maximize efficacy and minimize adverse effects. In-silico digital twins have emerged as a promising strategy to realize the vision of precision cardiology. While there is no uniform definition of a digital twin, it typically employs digital tools, including simulations of mechanistic computer models, based on patient-specific clinical data to understand arrhythmia mechanisms and/or make clinically relevant predictions. Digital twins have become part of routine clinical practice in the setting of interventional cardiology, where commercially available services use digital twins to non-invasively determine the severity of stenosis (computed tomography-based fractional flow reserve). Although routine clinical application has not been achieved for cardiac arrhythmia management, significant progress towards digital twins for cardiac electrophysiology has been made in recent years. At the same time, significant technical and clinical challenges remain. This article provides a short overview of the history of digital twins for cardiac electrophysiology, including recent applications for the prediction of sudden cardiac death risk and the tailoring of rhythm control in atrial fibrillation. The authors highlight the current challenges for routine clinical application and discuss how overcoming these challenges may allow digital twins to enable a significant precision medicine-based advancement in cardiac arrhythmia management.

## Abstract (de)

Herzrhythmusstörungen gehören nach wie vor zu den Hauptursachen für Morbidität und Mortalität. Aktuelle antiarrhythmische Therapien sind nur begrenzt wirksam, zum Teil aufgrund ihrer unspezifischen Wirkmechanismen. Die Präzisionsmedizin zielt darauf ab, für jeden Patienten eine maßgeschneiderte Therapie zu entwickeln, welche die Wirksamkeit optimiert und gleichzeitig die Nebenwirkungen minimiert. Eine vielversprechende Strategie, um diese Vision zu verwirklichen, sind digitale Zwillinge. Für diesen Begriff gibt es keine einheitliche Definition, gewöhnlich beinhaltet er aber Simulationen mechanistischer Computermodelle, die auf patientenspezifischen Daten basieren. Mit diesen Tools können Arrhythmiemechanismen analysiert und klinisch relevante Vorhersagen getroffen werden. Digitale Zwillinge sind in der interventionellen Kardiologie bereits etabliert, z. B. in kommerziellen Anwendungen, um den Schweregrad einer Stenose nichtinvasiv zu bestimmen (Computertomographie-gestützte fraktionelle Flussreserve). Für die Behandlung von Herzrhythmusstörungen haben sich bislang noch keine routinemäßigen klinischen Anwendungen digitaler Zwillinge etabliert. Jedoch wurden in den letzten Jahren erhebliche Fortschritte in diesem Bereich erzielt. Große technische und klinische Herausforderungen bleiben aber bestehen. In der vorliegenden Übersichtsarbeit geben wir einen kurzen Überblick über die Entwicklung digitaler Zwillinge in der kardialen Elektrophysiologie und stellen aktuelle Anwendungen vor, die das Risiko eines plötzlichen Herztods vorhersagen und eine individuelle Rhythmuskontrolle bei Vorhofflimmern erlauben. Wir erläutern die aktuellen Herausforderungen für die klinische Praxis und zeigen, wie digitale Zwillinge durch die Überwindung dieser Herausforderungen in der Zukunft einen bedeutenden Fortschritt in der präzisionsmedizinischen Behandlung von Herzrhythmusstörungen ermöglichen können.

## Introduction

Cardiac arrhythmias remain a major cause of death and disability [27]. For example, ventricular tachyarrhythmias are a major cause of sudden cardiac arrest (SCA) and subsequent sudden cardiac death, which represents ~50% of all cardiovascular deaths [30]. Similarly, atrial fibrillation (AF) affects more than 45 million people worldwide and is associated with 20–30% of all ischemic strokes, a 10–40% annual hospitalization rate, and a 1.5- to 3.5-fold increased risk of death [16]. Despite significant advances in diagnosis and treatment options, current management of cardiac arrhythmias is unable to prevent these adverse outcomes.

The limited efficacy of current antiarrhythmic therapies is likely in part attributable to a one-size-fits-most approach that ignores the diversity in arrhythmogenic risk and mechanisms. Growing awareness of this limitation has motivated the vision of precision cardiology, which aims to provide tailored therapies that maximize the efficacy and efficiency of healthcare delivery while minimizing adverse effects [10]. Precision medicine is enabled by the growing ability to obtain patient-specific information on disease processes through genetic, ‘omic,’ biomarker, and imaging methodologies. However, integrating the data generated by these diverse methodologies to guide therapy is highly challenging. In-silico ‘digital twins’ have emerged as a promising strategy to realize the vision of precision cardiology [10].

The present narrative review describes the background of digital twins and summarizes the state of the art. In addition, the authors highlight key challenges and future potential for the clinical application of digital twins in the field of cardiac electrophysiology.

## What are digital twins?

Computer models of cardiac electrophysiology have been around for many decades and have grown increasingly sophisticated [15, 28]. Several different types of computer models exist. The significant increase in computational resources and the staggering expansion of the amount of available data have enabled the development of advanced *data-driven models* [11, 15]. These models include classical risk scores derived from multivariable statistics, e.g., CHA_2_DS_2_-VASc (congestive heart failure, hypertension, age ≥75 [doubled], diabetes, stroke/thromboembolism [doubled], vascular disease, age 65 to 74, and sex category [female]), as well as artificial intelligence- or machine learning-based models [19]. These are able to map patient-specific input parameters to clinically relevant output parameters, but are typically static and do not explicitly integrate pathophysiological mechanisms, reducing their ability to generalize beyond the training space and limiting their explainability.

By contrast, *mechanistic models* integrate experimental data with biophysical principles to enable dynamic simulations of cardiac electrophysiology at different scales, e.g., from the opening/closing of individual ion channels to complex patterns of electrical (re-)excitation at the organ level. In recent years, these models have started to have real-world impact. For example, computer models play a central role in modern safety assessment of new pharmacological agents through the comprehensive in vitro proarrhythmia assay (CiPA) initiative [14], thereby potentially influencing commercial decisions on compound selection. In recent years, several cardiomyocyte models based on data from non-diseased human hearts, as well as models representing specific pathologies such as hypertrophic cardiomyopathy, heart failure, or atrial fibrillation have been developed [28]. Since cardiac arrhythmias are intrinsically multi-cellular phenomena, these models have been employed in multi-scale organ-level simulations to study the determinants of arrhythmia initiation and stability [28]. Since the early 2000s, organ-level models based on patient-specific anatomies have been developed [24] and have subsequently been further personalized with structural remodeling derived from non-invasive imaging data [2].

**Fig. 1:**
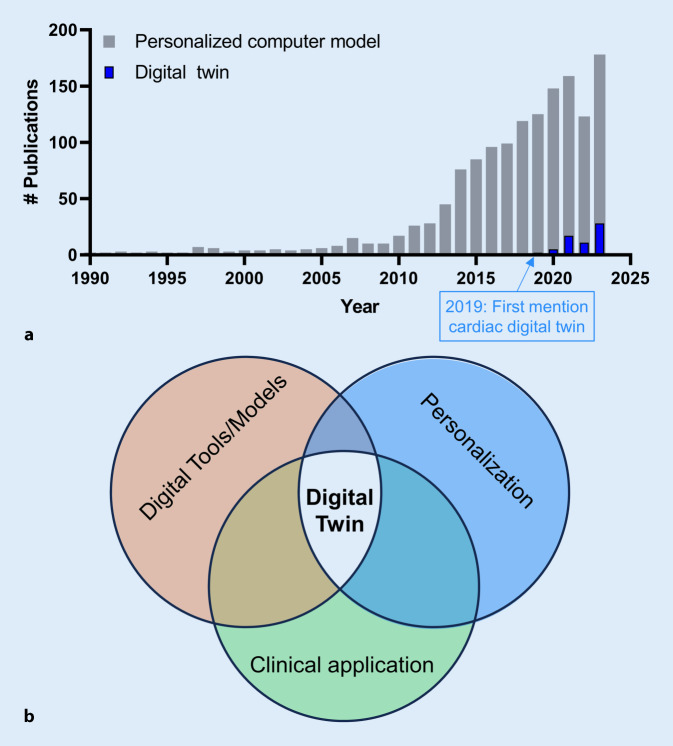
**a** Number of publications on personalized computer modeling (*gray bars*) and digital twins (*blue bars*) in PubMed from 1990 up to 2023. **b** Key components of a digital twin

Recently, hybrid approaches have emerged, such that the categories of data-driven vs. mechanism-based models represent a continuum, although digital twins typically tend towards the mechanism-based end of the spectrum. In 2019, the term “digital twin” appeared for the first time in PubMed in conjunction with the term “cardiac” (Fig. 1a). At present, there is no precise definition that sets digital twins apart from other (personalized) computer modeling efforts that have been around for many years. Previous definitions include: “a comprehensive, virtual tool that integrates coherently and dynamically the clinical data acquired over time for an individual using mechanistic and statistical models” [10]; “digital replicas of patient hearts derived from clinical data that match like-for-like all available clinical observations” [13]; “real-time digital replica of the individual patient’s heart, which is iteratively updated with up-to-date clinical data. Importantly, there is a two-way communication between the patient and the digital twin” [11]; and “a digital representation of organs or even patients, using tools capable of simulating personal health conditions and predicting patient or disease trajectories based on relationships learned both from data and from biophysics knowledge” [29]. From these and other studies, three key components emerge: (1) the use of digital tools and computer models; (2) the incorporation of patient-specific clinical data; and (3) deriving clinically relevant predictions (Fig. 1b). For all three components, significant challenges have been overcome to arrive at the current state of the art in digital twins for cardiac arrhythmia management, while numerous issues and opportunities remain. These topics are addressed in the remainder of this article, focusing primarily on digital twins employing mechanistic models.

## Current state of the art

**Fig. 2:**
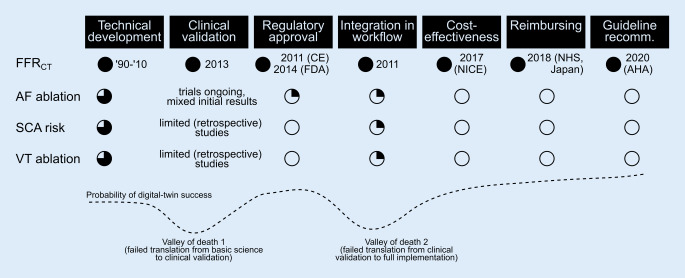
The pathway to digital-twin based solutions to personalize treatment guidance, illustrated for computed tomography fractional flow reserve (*FFR*_*CT*_), atrial fibrillation (*AF*) ablation, sudden cardiac arrest (*SCA*) risk prediction and ventricular tachycardia (*VT*) ablation. Progress is indicated with *circles*, with *open circles* indicating not yet attained, *filled circles* indicating fully realized, and *partially filled circles* reflecting the progress in each domain

Within the domain of cardiology, computed tomography (CT) fractional flow reserve (FFR_CT_) is among the most established clinical applications of digital twins. FFR_CT_ integrates patient-specific information about coronary anatomy obtained from CT in a computational model of fluid dynamics to simulate coronary blood flow and determine the functional significance of a lesion [18]. The computational methodology for this approach was developed around the turn of the century and has subsequently been validated against invasive recordings in numerous clinical studies and randomized controlled trials (RCTs) [26]. The approach has been commercialized, received CE Mark and Food & Drug Administration clearance, and is recommended by entities such as the National Institute of Health and Care Excellence for the (cost-)effective treatment of patients with coronary artery disease, successfully covering all aforementioned components (Fig. 2).

By contrast, digital twins for cardiac electrophysiology and arrhythmia management have not yet reached routine clinical adoption. Previous studies have shown how patient-specific anatomies and structural remodeling (fibrosis) can be obtained from non-invasive imaging [2]. Here, personalization typically entails creating a 3D mesh of a patient’s heart from imaging, including regions of scar and border zone, and using a computational electrophysiology model that includes pre-defined parameters for scar tissue, border zone tissue, and healthy tissue [28].

These patient-specific modeling frameworks have been applied retrospectively in all domains of cardiac arrhythmia management. For example, it has been shown that virtual arrhythmia risk prediction based on cardiac magnetic resonance (CMR) imaging-derived patient-specific models providesbetter stratification of SCA risk than left ventricular ejection fraction in 41 patients with ischemic cardiomyopathy [1]. Naturally, if one can predict the occurrence of arrhythmias, one can also evaluate how electrophysiological changes would modulate this inducibility, providing patient-specific treatment guidance. This has been done in retrospective studies for catheter ablation of infarct-related ventricular tachycardia [4, 22] and persistent AF [17], as well as inherited arrhythmia syndromes [28, 29] and resynchronization therapy [20].

Feasibility studies for the prospective use of computationally guided personalized ablation in ventricular tachycardia (*n* = 5) and AF (*n* = 10) have also been performed [5, 22]. However, to date, only a few small-scale single-centre RCTs have been performed. The first prospective study randomized 108 patients to routine clinical care (empirical ablation; primarily pulmonary vein isolation, PVI) or simulation-guided AF ablation evaluating a number of pre-defined ablation strategies in CT-derived patient-specific anatomical models. Although this study showed that it is feasible to tailor ablation therapy based on digital twins in clinical practice, there was no difference in outcome despite the use of significantly more extensive lesion sets in the simulation-guided group [25]. More recently, a prospective multi-center RCT, randomizing 170 patients with persistent AF to PVI plus empirical or PVI plus simulation-guided ablation, provided the first demonstration that computational modeling-guided ablation improved the rhythm outcome in persistent AF [3]. A third RCT comparing PVI with additional ablation guided by CMR-derived patient-specific models [5] is currently ongoing (ClinicalTrials.gov NCT04101539). No clinical trials are currently registered for simulation-guided treatment of ventricular arrhythmias or prevention of sudden cardiac death.

## Challenges and opportunities for digital twins of cardiac electrophysiology

### Challenges and opportunities related to model development

Cardiac arrhythmogenesis is an inherently multi-scale phenomenon. Dysfunction of specific ion channels or abnormalities in subcellular Ca^2+^ handling have been causally linked to arrhythmias in vivo and have been investigated in detailed computational cardiomyocyte models. However, such (sub)cellular models are computationally demanding, precluding their use in organ-level simulations required for investigating the proarrhythmic consequences of subcellular alterations or the complex pharmacodynamic effects of antiarrhythmic drugs. Recent work has shown how detailed cellular models can be used to parameterize simpler models that can be used in tissue-level simulations [6, 9], providing examples of how challenges related to multi-scale simulations can be partially overcome.

Another significant challenge for progress in the clinical translation of digital twins is the reproducibility and technical validation of the numerous different frameworks and implementations maintained by different researchers worldwide. While the sharing of models has improved through simulation frameworks such as Myokit [7] and OpenCARP [21], modeling standards such as CellML, and open source pipelines for mesh generation from imaging data [23], the fact that many models are still only available in custom-made, often poorly documented code, or that exact mesh details and initial conditions are often not reported, hinders the reproducibility and technical validation of model implementations.

### Challenges and opportunities related to personalization

Precision cardiology aims to deliver targeted, mechanism-based treatment for an individual patient. Until now, most digital twin studies have used non-invasive imaging data (CT, CMR) to obtain patient-specific anatomies and indicators of structural remodeling (generally fibrosis obtained through late gadolinium-enhanced CMR) [28]. However, existing modalities have important limitations, e.g., related to spatial resolution, imaging artefacts due to implanted devices, and standardization of cut-off values, leading to overestimation of fibrotic content and inability to identify all channels of surviving myocardium in a complex scar [12]. Recent advances, including dedicated high-resolution CMR protocols and photon-counting CT, may help to address some of these limitations.

Compared to structural remodeling, personalization of electrical parameters is less developed in most digital twin approaches. Standard 12-lead electrocardiograms (ECGs) are available for most patients and can be used to parameterize digital twins [13]. However, standard ECGs provide limited information on spatial repolarization patterns, resulting in a non-unique relationship between ECG and spatiotemporal electrophysiological properties, limiting the identifiability of model parameters. Invasive mapping data can be used to further personalize the electrophysiological properties of digital twins [23], but require an invasive procedure with data points collected over multiple heart beats and with limited spatial relation to the imaging-based mesh. Non-invasive electrocardiographic imaging may reveal local repolarization abnormalities that are not visible on a standard 12-lead ECG, e.g., in patients with idiopathic ventricular fibrillation [8], potentially providing a non-invasive data source to personalize model parameters. However, its spatial accuracy is limited and primarily reflects epicardial information. Importantly, none of these approaches provides clear information on the underlying biophysical mechanisms, with multiple combinations of ionic changes potentially giving rise to the same electrophysiological phenotype at baseline, but responding differently during tachyarrhythmias. Since molecular or functional data at the level of the cardiomyocyte are not available in the vast majority of patients, no truly personalized cellular electrophysiology models are currently available. Perhaps future advances, e.g., in the generation of patient-specific induced pluripotent stem cell-derived cardiomyocytes, may enable additional personalization of electrophysiological parameters, although numerous limitations remain, including functional immaturity and the inability to control the regional differences in electrophysiological properties (e.g., apicobasal or transmural).

Finally, even if high-quality patient-specific data can be obtained for model personalization, these data are typically only available at a single moment in time, ignoring the dynamic nature of cardiac electrophysiology and the sudden occurrence of arrhythmias. Recent work has shown how the performance of a static machine learning-based VT risk predictor derived from the baseline ECG dropped over time, whereas a dynamic model incorporating time-varying ECG data showed increased performance over time [19]. More research is needed to identify methodologies to incorporate dynamic changes in arrhythmogenic risk in digital twins, e.g., based on blood biomarkers or wearables.

### Challenges and opportunities related to clinical predictions and implementation

Ultimately, the proverbial proof of the pudding that will determine the clinical success of digital twins for cardiac arrhythmia management will be high-quality large-scale RCTs demonstrating the benefit of digital twin-guided care over routine clinical care on clinically relevant outcomes [12]. However, such trials are complex and time consuming, requiring close collaboration between clinicians, computational modelers, and industry [12, 15]. Even if such trials were to show positive results, significant efforts would be required to enable routine clinical application of digital twins, e.g., in terms of work-flow automation, standardization, and processing times, in order not to disrupt normal clinical care. On the other hand, digital twins based on mechanistic models have the advantage of being easily interpretable, potentially facilitating acceptance of model-guided care over other black-box approaches. Ultimately, the feasibility and cost-effectiveness of the use of digital twins for the treatment of the large number of patients with cardiac arrhythmias will need to be demonstrated to ensure reimbursement and subsequent routine clinical application (Fig. 2).

## Conclusions

The routine clinical application of digital twins for cardiac electrophysiology and arrhythmia management has never been closer. Advances in model development and personalization driven by advances in cardiovascular imaging, clinical electrophysiology, and digital/mobile health have opened up paths to retrospective validation of the clinical predictions of digital twins for all domains of cardiac arrhythmia management. In turn, these results have enabled the first prospective RCTs for AF ablation, demonstrating the feasibility of digital twin-guided care. However, this does not mean that the problem has been solved. Numerous challenges in model development, personalization, and clinical implementation still need to be overcome before true guideline-changing trials can be initiated.

## Declarations

For this article no studies with human participants or animals were performed by any of the authors. All studies mentioned were in accordance with the ethical standards indicated in each case.
